# The function of BK channels extracted and purified within SMALPs

**DOI:** 10.1042/BCJ20210628

**Published:** 2022-08-05

**Authors:** Jaimin H. Patel, Naomi L. Pollock, Jacqueline Maher, Alice J. Rothnie, Marcus C. Allen

**Affiliations:** 1 College of Health & Life Sciences, Aston University, Aston Triangle, Birmingham B4 7ET, U.K.; 2 School of Biosciences, University of Birmingham, Birmingham, U.K.; 3 Centre for Stress and Age-Related Disease, School of Pharmacy and Biomolecular Sciences, University of Brighton, Brighton, U.K.

**Keywords:** BK channel, detergent-free, planar lipid bilayer, SMA–PAGE, SMALP

## Abstract

Human BK channels are large voltage and Ca^2+^-activated K^+^ channels, involved in several important functions within the body. The core channel is a tetramer of α subunits, and its function is modulated by the presence of β and γ accessory subunits. BK channels composed of α subunits, as well as BK channels composed of α and β1 subunits, were successfully solubilised from HEK cells with styrene maleic acid (SMA) polymer and purified by nickel affinity chromatography. Native SMA–PAGE analysis of the purified proteins showed the α subunits were extracted as a tetramer. In the presence of β1 subunits, they were co-extracted with the α subunits as a heteromeric complex. Purified SMA lipid particles (SMALPs) containing BK channel could be inserted into planar lipid bilayers (PLB) and single channel currents recorded, showing a high conductance (≈260 pS), as expected. The open probability was increased in the presence of co-purified β1 subunits. However, voltage-dependent gating of the channel was restricted. In conclusion, we have demonstrated that SMA can be used to effectively extract and purify large, complex, human ion channels, from low expressing sources. That these large channels can be incorporated into PLB from SMALPs and display voltage-dependent channel activity. However, the SMA appears to reduce the voltage dependent gating of the channels.

## Introduction

**Figure 1.**
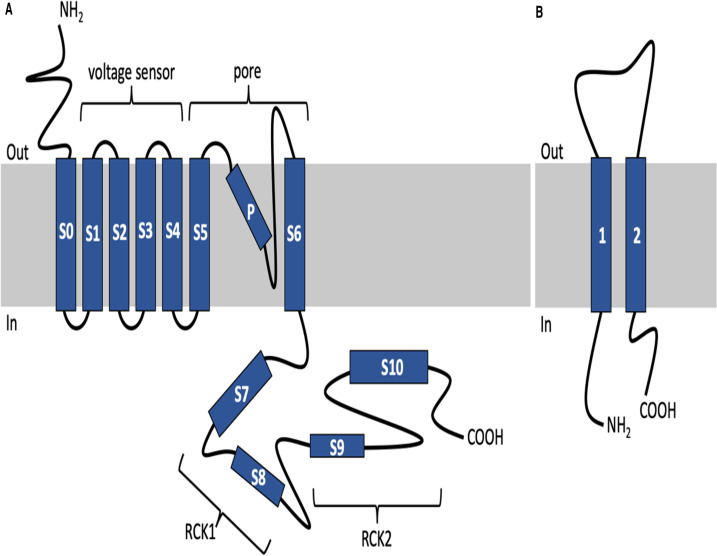
Topology of the BKα (A) and BKβ (B) subunits. The voltage sensor is formed by S1–S4; the pore is formed by S5, P and S6; the regulator of potassium conductance domains 1 and 2 are formed of S7–8 and S9–10, respectively.

The voltage and Ca^2+^-activated K^+^ channels, known as: BK (Big Potassium); maxi-K; slo1, Kca1.1 or KCNMA1/2, are a group of potassium channels characterised by their ability to conduct potassium ions through the cell membrane with impressive selectivity. BK channels have an extremely large conductance of ∼260 pS and activate in response to membrane depolarisation and binding of intracellular Ca^2+^ and Mg^2+^ [1,2]. BK channels are vital in the regulation of many important processes: contraction of smooth muscle; neurotransmitter release; potassium secretion in the kidney and hearing [3]. The core channel consists of four pore-forming α subunits that are encoded by a single Slo1 gene (Slowpoke or [KCNMA1]) (Figure 1A). The function of BK is modulated by two main factors: intracellular Ca^2+^ concentration and membrane potential. However, the alternative splicing of Slo1 during mRNA translation and subsequent modulation by accessory β subunits gives rise to a very wide functional diversity [2,4,5]. There are four types of β subunit, each with a defined tissue specific expression profile, that distinctly alter the gating properties of the core channel. Beta subunit incorporation is tissue specific: β4 is expressed exclusively in the brain; β2 and β3 are also highly expressed in the brain whereas β1 is expressed primarily in smooth muscle [6]. In addition, four extra gamma subunits and their many splice variants were discovered recently [7,8], which provide further diversity. The core α tetramer channel can associate with up to 4 β subunits and it's proposed these would affect the channel's gating [9].

The sheer size of the BK channel can make it difficult to study or manipulate. The core channel consists of a tetramer of four 125 kDa α subunits. Each monomer has seven transmembrane regions (Figure 1A), and thus**,** the tetramer will have a size of ∼500 kDa and consist of 28 transmembrane domains. The channel can then associate with up to four β subunits, each subunit being ≈21 kDa in weight with two transmembrane domains, giving the channel complex a potential size of 584 kDa with up to 36 transmembrane domains (Figure 1). This is a large multimeric complex, which has proved tricky to solubilise intact using detergents. For the recent cryo-EM structure of BK channel, the α subunit was truncated at the C-terminus to improve stability, and during purification cholesterol hemisuccinate and lipids had to be added [10]. In recent years, the development of SMA (styrene maleic acid) polymer as an alternative to detergents for membrane protein solubilisation has shown great promise for providing greater stability to membrane proteins [11–14]. Whereas detergent can disrupt protein:protein interactions, SMA often extracts complexes intact [15–17]. The SMA polymer inserts into the membrane and forms small discs of lipid bilayer encircled by the polymer, termed SMALPs (SMA lipid particles) [18]. The approach facilitates the extraction of membrane proteins whilst retaining the lipid bilayer environment known to be important for the function of many membrane proteins. The particles formed are small, stable, and amenable to many downstream techniques [11–15,18–22]. SMA has been successfully used to extract and purify ion channels (KscA and ROMK) for functional study previously [13,23], but these channels are significantly smaller and less complex than BK. Therefore, we investigated the extraction of human BK channel using SMA polymer, and subsequently the function of purified BK when inserted into planar lipid bilayers (PLB).

## Methods

### Cell culture and membrane preparation

HEK293 cells, stably expressing hSloα1 [24], were cultured in Dulbecco's modified Eagle Media supplemented with 10% (v/v) foetal bovine serum, non-essential amino acids, 5 µg/ml Blasticidin S (Sigma), 100 U/ml penicillin and 100 mg/ml streptomycin. HEK293 cells, stably expressing hSloβ1 in addition to hSloα1 were grown as above, with the addition of 1 mg/ml Geneticin (Sigma) [24]. Cells were harvested at confluency from triple layered T175 flasks using non-enzymatic cell dissociation media.

Cells were pelleted by centrifugation (1000***g***, 10 min, 4°C) and resuspended in buffer 1 (50 mM Tris, pH 7.4, 250 mM sucrose and 0.25 mM CaCl_2_) supplemented with protease inhibitors (1 µM pepstatin, 1.3 µM benzamidine and 1.8 µM leupeptin). Cells were lysed by nitrogen cavitation (500 psi, 15 min, 4°C) and cell debris removed by low-speed centrifugation (750***g***, 20 min, 4°C). Membranes were harvested by ultracentrifugation (100 000***g***, 20 min, 4°C), and resuspended in buffer 2 (20 mM Tris pH 8 and 150 mM NaCl) at 60 mg/ml (wet pellet weight). Aliquots were stored at −80°C for up to 6 mo.

### Solubilisation and purification

SMA2000 polymer from Cray Valley was hydrolysed and prepared as described previously [25,26]. Membranes from BK expressing HEK293 cells were mixed with an equal volume of 5% (w/v) SMA2000 in buffer 2, to give a final concentration of 30 mg/ml membrane and 2.5% (w/v) SMA and shaken gently at room temperature 1 h. The soluble and insoluble phases were separated by ultracentrifugation (100 000***g***, 20 min, 4°C). The insoluble pellet was resuspended in an equal volume of buffer 2 supplemented with 2% (w/v) SDS. Samples were analysed by western blotting, using either an anti-his primary antibody (R&D Systems) with anti-mouse HRP secondary antibody (Cell Signalling), or an anti-hsloα primary antibody (Abcam, ab3586) or anti-hsloβ primary antibody (Abcam, ab3587), with anti-rabbit HRP secondary antibody (Cell signalling). Blots were imaged by chemiluminescence (Pierce) using a C-Digit Western blot scanner (Licor), and efficiency of solubilisation was quantified by densitometry (ImageJ).

The soluble fraction was incubated with HisPur Ni-NTA resin (ThermoFisher) bed volume (bv) of 100 µl resin/ml of soluble protein, rotating at 4°C overnight. The resin and sample were poured into an empty gravity flow column and the flow through collected. The resin was washed five times with 10 bv of buffer 2 supplemented with 20 mM imidazole and twice with 10 bv of buffer 2 supplemented with 40 mM imidazole, before elution (5 × ½ bv) with buffer 2 supplemented with 200 mM imidazole. Fractions were analysed by SDS–PAGE followed by silver staining (Pierce). Elution fractions containing purified BK channel were pooled and analysed by western blotting using anti-his, anti-hsloα or anti-hsloβ primary antibodies. Purified protein could be concentrated using filter concentrators (with a molecular weight cut off of 100 kDa), and protein concentration determined using BSA standard SDS–PAGE as described previously [26]. Pure proteins within SMALPs were stored at 4°C for up to one week or at −80°C for long-term storage.

### SMA–PAGE

Native SMA–PAGE analysis of the purified protein samples was based upon previously published methods [27]. Briefly, samples of membranes or pure protein were mixed with a native loading dye (50 mM Tris pH8, 0.5 mg/ml bromophenol blue, 25% glycerol) at a ratio of 4 : 1. These were loaded into Tris/glycine gels with an acrylamide gradient of 4–16% (w/v) (Bio-Rad, U.K.) alongside molecular weight markers, both native and denaturing (NativeMARK, ThermoFisher U.K.; peqGOLD Protein Marker VI; VWR, U.K.). Proteins were resolved by electrophoresis at 150 V for 1 h at room temperature in a buffer of 25 mM Tris, 192 mM glycine. Before transfer, native gels were incubated with denaturing running buffer (25 mM Tris, 192 mM glycine, 0.1% SDS) to improve the efficiency of transfer. Proteins were transferred from these gels to nitrocellulose membrane for immunoblotting (7 min at 20 V, iBlot2 dry blotting system, ThermoFisher, U.K.). Thereafter the immunoblotting process was as described above, and blots were imaged using enhanced chemiluminescence reagents (Cytiva, U.K.) and a digital imaging system (Amersham Imager 600, Cytiva, U.K.). The native markers were not prestained or visible by chemiluminescence so the relative migration of the native and denaturing markers on the stained gels were used to infer the molecular weights of the test samples on immunoblots.

### Electron microscopy

Copper electron microscopy grids with a formvar-carbon support film (Agar Scientific) were glow discharged for 2 min to distribute negative charge on the surface. Purified BKα+β1 within SMALPs (10 µl at 2 µg/ml) was placed on each grid and incubated for 2 min at room temperature. Excess solution was blotted off and grids washed three times with distilled water before gently drying using Whatman filter paper. Uranyl acetate stain (3 µl drop of 2% (w/v)) was added to each grid and incubated for 4 min at room temperature, followed by three washes with distilled water. The grids were visualised using a JEOL 2100Plus super high resolution TEM-STEM at room temperature and images were acquired at 60 000× (Warwick University Imaging Facility).

### Planar lipid bilayer formation and electrophysiology

Planar lipid bilayer recordings were carried out as described previously [9,24,28]. Briefly, lipids were dissolved in n-decane at a concentration of 15 mg/ml. Two lipid mixtures were used the first consisted of a (1 : 1 ratio) 1-palmitoyl-2-oleoyl-sn-glycero-3-phosphoethanolamine (POPE, Avanti Polar Lipids) and 1-palmitoyl-2-oleoyl-sn-glycero-3-phospho-L-serine (sodium salt) (POPS, Avanti Polar Lipids). The second consisted of positively charged lipids composed of POPE and 1-palmitoyl-2-oleoyl-sn-glycero-3-phosphocholine (POPC, Avanti Polar Lipids, 4 : 1 ratio).

To construct a bilayer, lipid mixture was drawn across a 0.25 mm diameter hole in a polystyrene cup which separated two solution filled chambers designated cis and trans (Warner Instrument's lipid bilayer chamber/cup, BCH-13A and CP13A-250). The cis chamber (to which SMALP–BK complexes were added) was grounded. Single channel currents between the two chambers were recorded on Bio-logic BLM-120 amplifier, sampled at 10 KHz using a CED 1401+ A–D converter and filtered at 1 KHz. Only bilayers that had a leak conductance of <10 pS and an initial capacitance of at least 150 pF were used. Recordings were made at a ±50 mV transmembrane potential, unless otherwise stated. Bilayers were bathed in symmetrical recording solutions containing 150 mM KCl; 10 mM HEPES pH 7.2, 1 mM EGTA, 1 mM MgCl_2_ and 1.05 mM CaCl_2_ to yield 50 µM free Ca^2+^ in the recording buffer. The orientation of the channel was determined by measuring the P_o_ at ±50 mV. If the channel inserted with correct orientation, then channels were opened by depolarisation, if channels inserted into the bilayer backwards then the channel was opened by hyperpolarisation. It does not matter which way the channels insert because the *cis* and *trans* recording solutions are identical.

### Electrophysiological analysis

Recordings (30–300 s) were analysed offline using WinEDR v2.3.9 software (Strathclyde electrophysiological software). Single current amplitudes were determined from the peaks of two Gaussian functions fitted to Patlak current amplitude histograms. The difference between the two Gaussian means yielded the single channel current from which the single channel conductance could be determined. The open probability (P_o_) was determined by fitting a Gaussian function to current amplitude histograms, integration of these histograms yielded the probability of a channel being open. Plots of voltage against single channel current were fit with a linear best fit line, and plots of voltage against P_o_ were fitted with a Boltzmann sigmoidal curve using GraphPad Prism.

## Results

### Extraction & purification of BK channels

**Figure 2.**
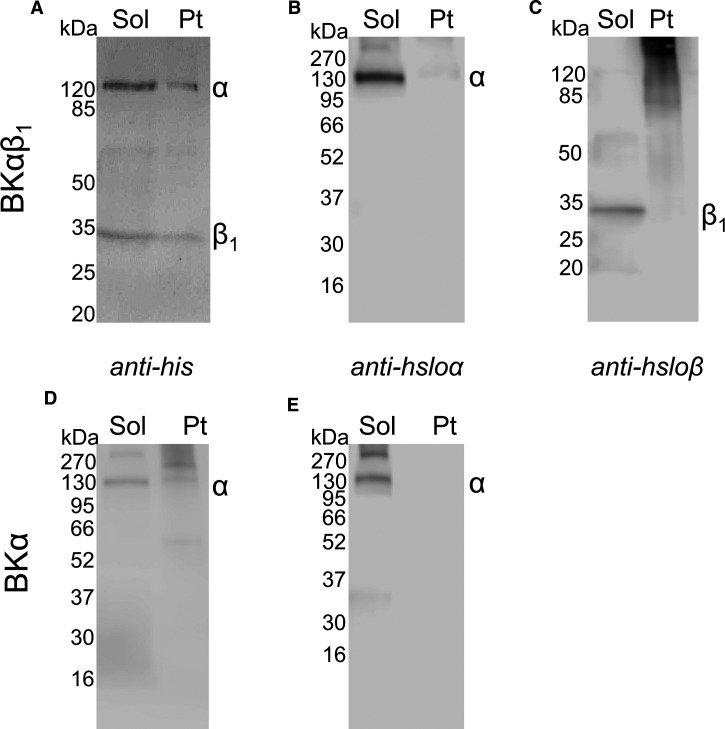
BK channel can be solubilised using SMA polymer. Membranes from HEK cells stably expressing BK channels (30 mg/ml wet pellet weight) were mixed with 2.5% (w/v) SMA2000 for 1 h at room temperature with shaking. Samples were subjected to ultracentrifugation (100,000***g***, 20 min, 4°C) and the supernatant containing soluble protein harvested (sol). The pellet containing insoluble material was resuspended in an equal volume of 2% (w/v) SDS (Pt). (**A**–**C**) Soluble and insoluble material from BKαβ_1_ membranes analysed by western blot using (**A**) an anti-his primary antibody to detect both the α and β_1_ subunits, (**B**) an anti-hsloα primary antibody and (**C**) using an anti-hsloβ primary antibody. (**D** and **E**) Soluble and insoluble material for BKα-only channels analysed by western blot using (**D**) an anti-his antibody or (**E**) an anti-hsloα antibody. Solubilisation efficiency was calculated by densitometric analysis (Image J) of anti-his western blots as shown in **A** & **D**.

**Figure 3.**
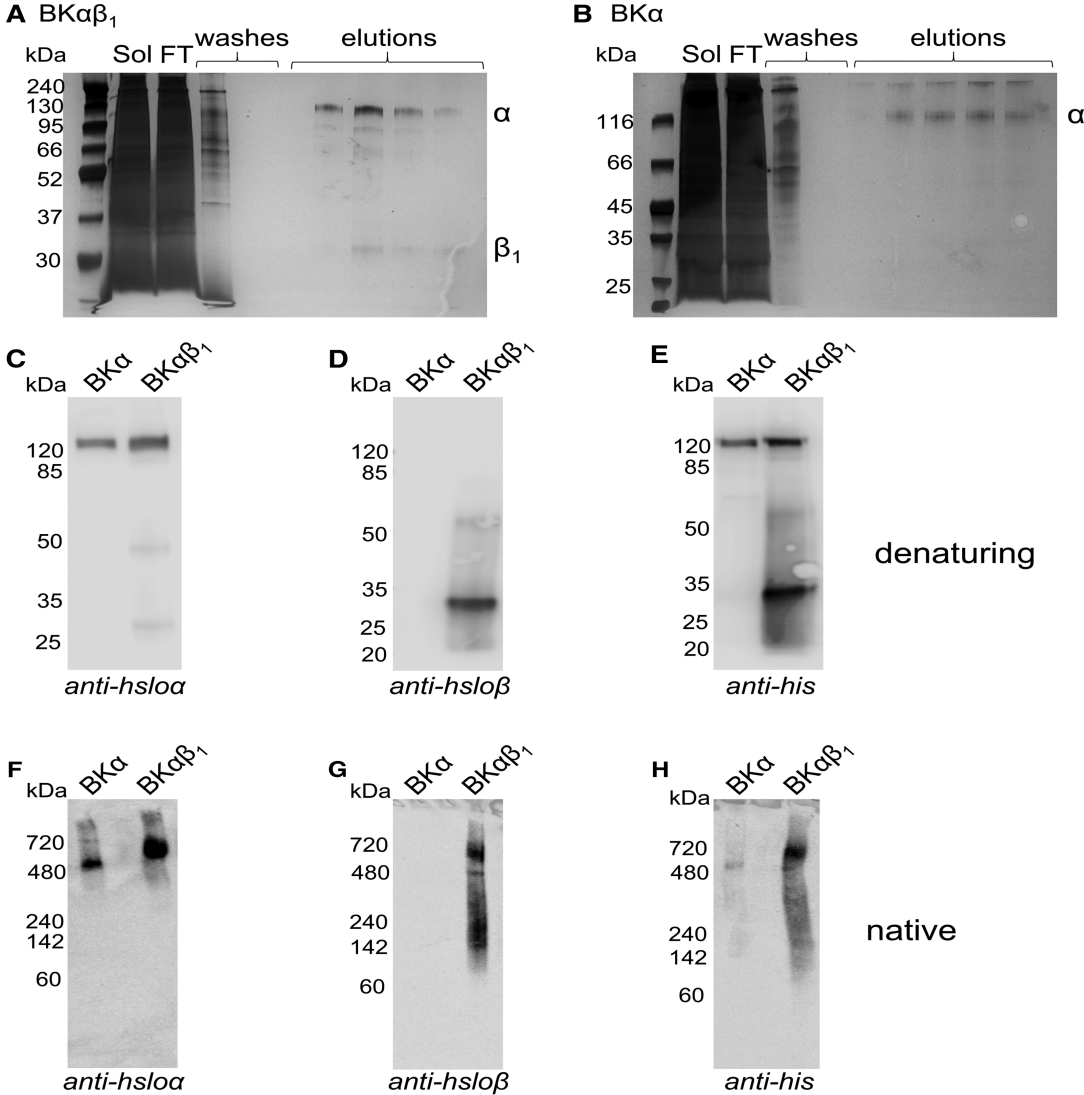
SMALP-encapsulated BK channels can be purified by affinity chromatography. (**A** and **B**) Silver stained SDS–PAGE showing the Ni-NTA affinity purification procedure. SMA-solubilised Bkαβ_1_ membranes (**A**) or BKα-only membranes (**B**) were mixed with HisPur resin overnight at 4°C, shaking. FT represents the flowthrough containing unbound protein. Five 10 bv washes were carried out with buffer containing 10 mM imidazole, and five ½ bv elution fractions obtained using buffer containing 200 mM imidazole. (**C**–**E**) Western blot analysis of purified BKα-only and BKαβ_1_ using (**C**) an anti-hsloα, (**D**) an anti-hsloβ or (**E**) an anti-his primary antibody. (**F**–**H**) Native SMA–PAGE western blots of purified BKα-only and BKαβ_1_ using (**F**) an anti-hsloα, (**G**) an anti-hsloβ or (**H**) an anti-his primary antibody.

Due to its large size, the first question was whether SMA could effectively extract BK from membranes. BK channels composed of α subunits only and channels composed of α and β1 subunits were stably expressed in HEK cells [24]. As shown in Figure 2, SMA was able to effectively solubilise α only and α + β1 channels. Densitometric analysis of the anti-his blots showed BKα had a solubilisation efficiency of 93 ± 3% (*n* = 4), whilst for the BKαβ1, 88 ± 5% (*n* = 4) of the alpha subunit was extracted and 75 ± 5% (*n* = 4) of the beta subunit was extracted. The solubilised BK channels were then purified by Ni-NTA affinity chromatography, as shown in Figure 3. The purification was optimised by varying parameters such as resin binding time and concentration of imidazole in the washes (Supplementary Table S1). Some contaminants can still be seen, but the most prominent bands correspond to the BK subunits. Purified SMALPs containing α + β1 BK channel were imaged by negative stain electron microscopy (Supplementary Figure S1) and showed particles of ∼17 nm in diameter.

Whilst we have shown that BK protein can be solubilised with SMA (Figure 2) and purified using affinity chromatography (Figure 3A–E) this does not prove that BK was isolated as a complete oligomeric ion channel complex i.e. tetramers or octamers. Therefore, we carried out native SMA–PAGE western blots on the purified proteins (Figure 3F–H). As can be seen in Figure 3F, the BKα-only sample shows a single band just above the 480 kDa marker. This corresponds well to a tetramer of alpha subunits (4 × 125 kDa), which is known to be the minimal functional channel. Furthermore, for the BKα + β1 samples the native PAGE shows a band at a higher molecular weight than the BKα alone. This band is detected by both the anti-hsloα and anti-hsloβ antibodies (Figure 3F,G), therefore showing that the alpha and beta subunits were co-extracted and purified as a complex.

### The function of the purified BK channels composed of α subunits

**Figure 4.**
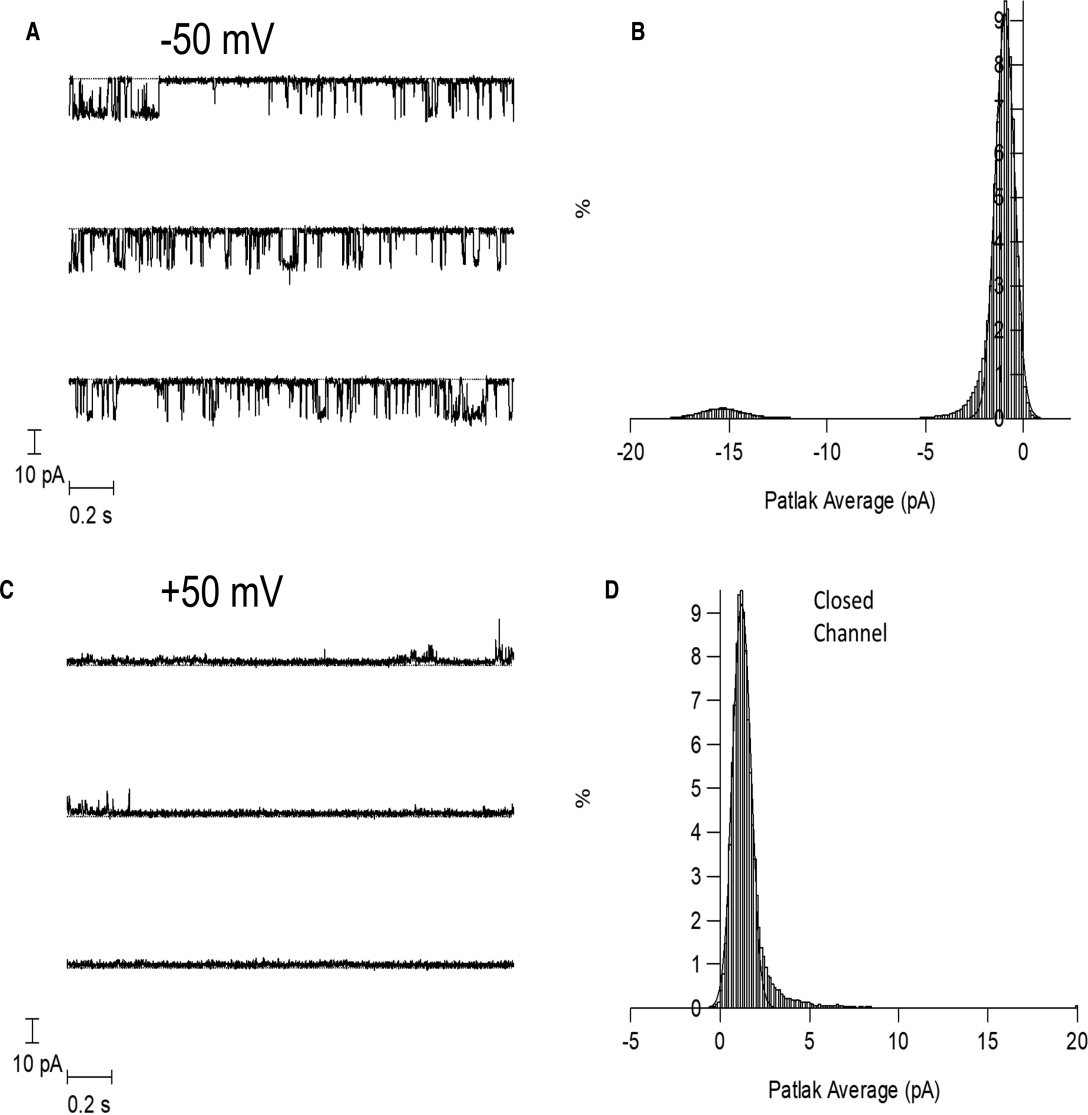
BK channels (hslo α) purified using SMALP's can form functional BK channels in planar lipid bilayers. The recordings are made at ±50 mV in the presence of 50 µM free Ca^2+^. The planar lipid bilayer was formed from 50 : 50 POPS and POPE. The BKα channel/SMALP complex has inserted into the bilayer backwards and C and O represent the closed and open state. (**A**) BK single channel currents at −50 mV. (**B**) The Patlak amplitude histogram of the potassium current flowing through the channel. The histogram has been fitted to two Gaussian curves. The open probability is ∼0.15. (**C**) BK single channel measurements at +50 mV. BK channel openings are extremely brief and do not achieve a full amplitude due to the filtering of the recording. (**D**) The Patlak running amplitude histogram of the potassium current flowing through the channel.

To further prove that SMA extracted complete and functionally intact ion channels we used the planar lipid bilayer technique to investigate channel gating of the solubilised and purified protein. Single channel currents were recorded from pore forming α subunits encapsulated within SMALPs and inserted into planar lipid bilayers (PLB). The SMALP–BKα complexes inserted into PLB with a clear preference for inserting backwards and the orientation could be determined by looking at the voltage sensitivity of channel opening. Figure 4 shows a single channel-complex that has inserted backwards into the PLB because the channel is opened by hyperpolarisation rather than depolarisation. At a voltage of −50 mV single channel openings can be seen, and the single channel conductance is normal at ∼280 pS. However, the open probability (P_o_) is only 0.15, which is lower than expected for a voltage of 50 mV and a free Ca^2+^ concentration of 50 µM. In our hands, membrane preparations containing BKα channels incorporated into planar lipid bilayers, under these conditions, would normally show an open probability of ∼0.9, thus the voltage sensitivity of the purified SMALP-channel complex seems impaired [9,24].

### The function of the purified BK channels composed of α and β1 subunits

**Figure 5.**
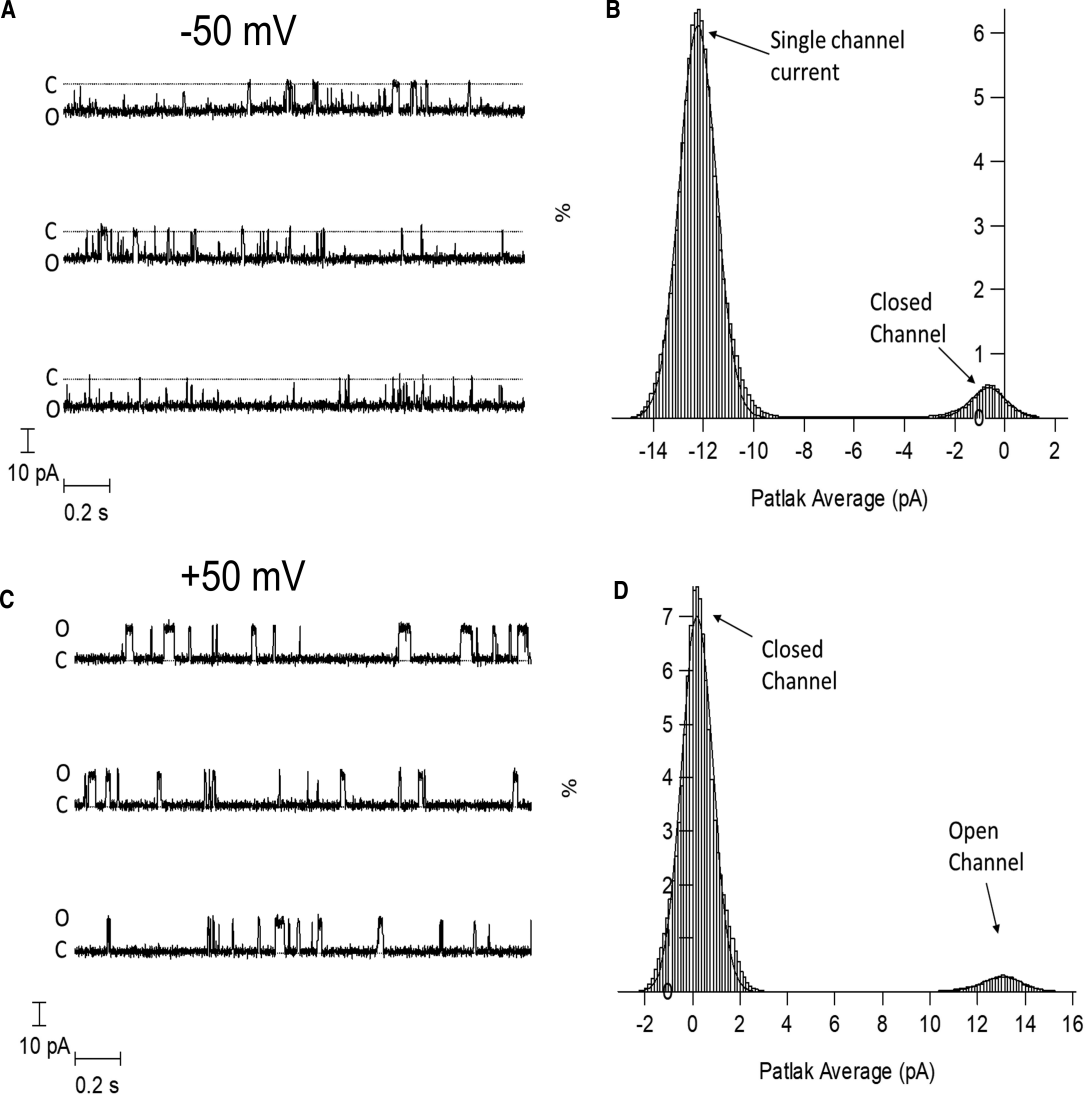
BK channels (hslo α + β1) purified using SMALP's can form functional BK channels in a planar lipid bilayer. The recordings are made at ±50 mV in the presence of 50 µM free Ca^2+^. The planar lipid bilayer was formed from 50 : 50 POPS and POPE. The BKα channel/SMALP complex has inserted into the bilayer backwards and C and O represent the closed and open state. (**A**) BK single channel currents at −50 mV. (**B**) The Patlak amplitude histogram of the potassium current flowing through the channel. The histogram has been fitted to two Gaussian curves. (**C**) BK single channel currents at +50 mV. (**D**) The Patlak amplitude histogram of the potassium current flowing through the channel at +50 mV.

Single channel currents were also recorded from pore forming α and β1 subunit purified within SMALPs and inserted into PLB. Again, the channel illustrated in Figure 5 has inserted backwards. The Patlak amplitude histograms reveal a normal single channel conductance of ≈257 pS. The presence of the β1 subunits increases the open probability at both ±50 mV, compared with α subunits alone, as would be expected (Figures 4, 5). However, the open probability is still lower than would be expected for a channel composed of both α and β1 subunits. At this voltage and calcium ion concentration, for un-purified channels inserted into PLB directly from membrane extracts, it would be expected that the channel is open ∼90% of the time at both depolarising and hyperpolarising potentials [9,24] (Supplementary Figure S2). When comparing the relationship between applied voltage and channel current we observe no differences between SMALP-purified BKαβ channels or those from crude membranes (Supplementary Figure S2B). However, examination of the open probability (P_o_) as a function of applied voltage shows a clear shift in the curve for SMALP-purified BKαβ channels compared with those from membrane preparations (Supplementary Figure S2C), suggesting that the voltage sensitive gating is impaired.

Having shown that SMALP–BK channel complexes can insert into neutral planar lipid bilayers, we also investigated positively charged bilayers composed of POPC and POPE (20 : 80). SMALP–BK channel complexes can also insert into these bilayers, and this is shown in Supplementary Figure S3. The channel inserted with a reverse orientation and opened on depolarisation. SMALP complexes carry a negative charge, and it was hoped that they would quickly insert into the positively charged PLB and disaggregate, releasing the encapsulated proteins and enabling normal channel gating. However, in this recording, the channel seems to remain for long periods in a sub conductance state, only rarely fully opening, again suggesting a restricted gating for the channel.

## Discussion

In this study, the use of SMA for extraction and purification of human BK channel was investigated. SMA was able to successfully extract both the α-only core BK channel, and the α + β1 channel from HEK293 cells with high efficiency. This extraction efficiency is much higher than a previous report which extracted the ion channels hKCNH5 and hKCNQ1 from Cos cells [29], however, that study extracted proteins from whole cells rather than membrane preparations, which may have affected the extraction efficiency. With between 28 and 36 transmembrane helices (TMs), BK channel is one of the larger proteins to have been extracted with SMA. Other large proteins reported include hKCNQ1 with 24 TM [29], AcrB with 36 TMs [30,31] and the SLAC channel with 30 TMs [32]. However, the largest complex extracted to date is the Alternative Complex III, which comprises 6 different subunits and 48 TMs [15]. Typically, the diameter of SMALPs is ∼10 nm, but when larger proteins/complexes are present it seems that larger particles can be formed. The particles formed in the current study were ∼17 nm in diameter, which agrees well with previously reported extraction and purification of the hKCNH5 ion channel [29]. One of the often-reported advantages of the SMA approach is the maintenance of the lipid bilayer around a protein, but with the larger proteins/complexes it is not clear how much lipid actually remains. However, cryo-EM structural studies have shown a belt of lipids around large proteins within the SMALP and have enabled the detection of specific lipid–protein interactions [15,31,32]. Investigation of the structure and lipid environment could be an interesting avenue to pursue with BK in the future.

SMALP encapsulated BK channel was purified by Ni-NTA affinity chromatography. Whilst the degree of purity achieved appears lower than that reported for many other proteins in SMALPs in the literature, it should be noted that the expression level of the channel was relatively low, given that these are stably transfected cell lines. Bada Juarez et al. [33] previously reported the extraction and purification of the dopamine receptor from stable HEK cells, achieving yields comparable to those we observe for BK. Interestingly, the SMALP encapsulated BK bound well to the affinity resin, with very little protein in the flow-through. This is an aspect often reported in the literature to be challenging with proteins within SMALPs [19,33,34]. Perhaps the fact that each subunit within the multimeric channel contained a his_6_-tag helped to increase the avidity of the resin binding.

To prove that the channel was being extracted and purified as a functional complex we examined the purified protein using native SMA–PAGE as well as monitoring the function of BK by inserting it into a PLB and recording the current. The native SMA–PAGE showed that BKα-only was extracted and purified as a complex >480 kDa, suggesting a tetrameric structure. When the β1 subunits were also present the complex extracted and purified was larger in size, and could be detected by both anti-hsloα and anti-hsloβ antibodies, showing co-extraction of the complex. The band for the complex containing beta subunits is larger and more ‘smeary’ than the alpha alone. This is likely because the alpha tetramer can associate with between 1 and 4 beta subunits [9]. When measuring function, as shown previously for the smaller ion channels KcsA and ROMK, it is possible to insert SMALP encapsulated BK proteins into a PLB simply by adding them to one side of the PLB and mixing [13,23]. Single channel opening and large conductances were observed, as expected for BK [9,24]. There was also a clear difference in the open probability observed for the channels from cells expressing the β1 subunit in addition to the core α subunit [9,24], again showing that the β1 subunits were indeed co-purifying with the core channel. One notable and useful observation is that we only ever observed insertion of a single channel. In contrast when using membrane extracts it can be common to see more than one channel inserting and that can complicate the analysis [24]. The ability to add the SMALP encapsulated protein directly to the PLB system following purification is an advantage over detergent mediated purification, where the detergent present would disrupt the bilayer. Krajewska and Koprowski [23] previously demonstrated that whilst free SMA polymer can disrupt the PLB, when added as part of a SMALP–protein complex the integrity of the bilayer was not affected.

SMALP-purified BK channel inserted into the PLB did demonstrate voltage dependent changes in open probability. However, higher membrane potentials were required for opening than is usually observed for BK. For example, at 50 mV in the presence of 50 µM Ca^2+^ for the α-only channel the open probability was only 0.15 compared with a typical open probability of 0.9 at this voltage (Figure 4) [9,24]. Similarly, for the α + β1 channels over 90% opening would normally be observed at both ±50 mV, and although we observed opening at both voltages, the open probability was greatly reduced at +50 mV (Figure 5 and Supplementary Figure S2). This suggests that the gating of the channel is impaired in some way. What is causing this is not entirely clear. One possibility is that the channel is being constrained. This is also suggested by the sub conductance states observed when the PLB was formed from positively charged lipids (Supplementary Figure S3). Perhaps the SMA polymer remains associated with the channel and/or lipids and causes this constraint. It is not known what happens to the SMA or where it goes when the SMALP inserts into the PLB. Similarly, several studies have reported the reconstitution of proteins from SMALPs into proteoliposomes, simply by mixing them [35–37], but it is not known what happens to the SMA in those circumstances. Why this gating problem is observed for BK, but not previously for KcsA or ROMK, isn't entirely clear, but this may relate to the size of the BK channels (11 × 13 × 13 nm channel dimensions) [38], which is even bigger when associated with beta sub units (15 × 15 × 15 nm channel dimensions) [10], and the limited lipid likely to be left around BK within the SMALP, in comparison KcsA, is much smaller (3.4 × 4.7 × 2.5 nm channel dimensions) [39]. In the recent structure of human BK channel, it was observed that lipid molecules make significant contributions to the α/β interfaces, suggesting they are an integral part of the BK channel complex, so SMA remaining associated with them may affect function [10]. It would be interesting in the future to investigate where the SMA goes and if/how it is causing the gating issues for BK. Similarly, it would be interesting to investigate if the same effect is observed with other polymer variants, such as DIBMA. DIBMA does not contain the aromatic styrene groups of SMA, and forms larger discs which would be expected to contain more lipid around BK.

In conclusion, we have demonstrated that SMA can be used to effectively extract and purify large, complex, human ion channels, from low expressing sources. These large channels can be incorporated into PLB from SMALPs and display voltage-dependent channel activity. However, the SMA appears to be affecting the gating of the channels.

## Data Availability

The underlying data for this study can be found at the Aston Explorer Data Repository (https://doi.org/10.17036/researchdata.aston.ac.uk.00000504).

## Open Access

Open access for this article was enabled by the participation of Aston University in an all-inclusive *Read & Publish* agreement with Portland Press and the Biochemical Society under a transformative agreement with JISC.

## Abbreviations

BK: Large conductance Ca^2+^ activated and voltage-sensitive K^+^ channel
bv: bed volume
HEK: human embryonic kidney cell line
HRP: horseradish peroxidase
Hsloα1: human slow poke alpha subunit K^+^ channel
hsloβ1: human slow poke regulatory beta one subunit
Ni-NTA: nickel nitrilotriacetic acid
PLB: planar lipid bilayer
POPC: palmitoyl-2-oleoyl-sn-glycero-3-phosphocholine
POPE: palmitoyl-2-oleoyl-sn-glycero-3-phosphoethanolamine
POPS: palmitoyl-2-oleoyl-sn-glycero-3-phosphoserine
SDS: sodium dodecyl Sulfate
SDS–PAGE: SDS polyacrylamide gel electrophoresis
SMA: styrene maleic acid
SMALP: SMA lipid particle
SMA–PAGE: SMA polyacrylamide gel electrophoresis
