# Supplementary material for: The function of BK channels extracted and purified within SMALPs
Source: Biochem J. 2022 Aug 5;479(15):1609–19. doi: 10.1042/BCJ20210628 (PMC9444072; doi:10.1042/BCJ20210628)
Supplement: Supplementary Material 1 [file BCJ-479-1609-s1.pdf]

Supplementary Information

The function of BK channels extracted and purified within SMALPs.

Jaimin H. Patel[1], Naomi L. Pollock[2], Jacqueline Maher[3], Alice J. Rothnie[1*] & Marcus C. Allen[3*]

[1]College of Health & Life Sciences, Aston University, Aston Triangle, Birmingham, B4 7ET, UK

[2]School of Biosciences, University of Birmingham, Birmingham, UK

[3]Centre for Stress and Age-Related Disease, School of Pharmacy and Biomolecular Sciences, University of Brighton, UK

Supplementary Table 1

Parameter varied		Findings	Chosen condition
Incubation time with Ni-NTA resin	4 hours vs overnight	Limited binding to the resin after 4 hours. Better binding after overnight incubation.	Overnight binding with shaking at 4°C.
Volume of Ni-NTA resin	100µl or 200µl Ni-NTA resin/ml solubilised membranes	Increasing the volume of Ni-NTA resin had no impact.	100µl Ni-NTA resin/ml membranes.
Concentration of imidazole in the washes<br>Volume/number of washes	20mM, 40mM or 60mM imidazole<br>1 bv, 5bv or 10bv<br>2 or 5 washes	60mM imidazole caused loss of significant amount of BK channel.<br>Better purity achieved with more washing.	5 x 10bv washes with 10mM imidazole + 2 x 10bv volumes washes with 20mM imidazole.

Supplementary Table 1. Optimisation of affinity purification for the BK channel. Summary of the parameters that were altered to optimise the purification of BK channels, the main findings and the final conditions selected.

Supplementary Figure 1

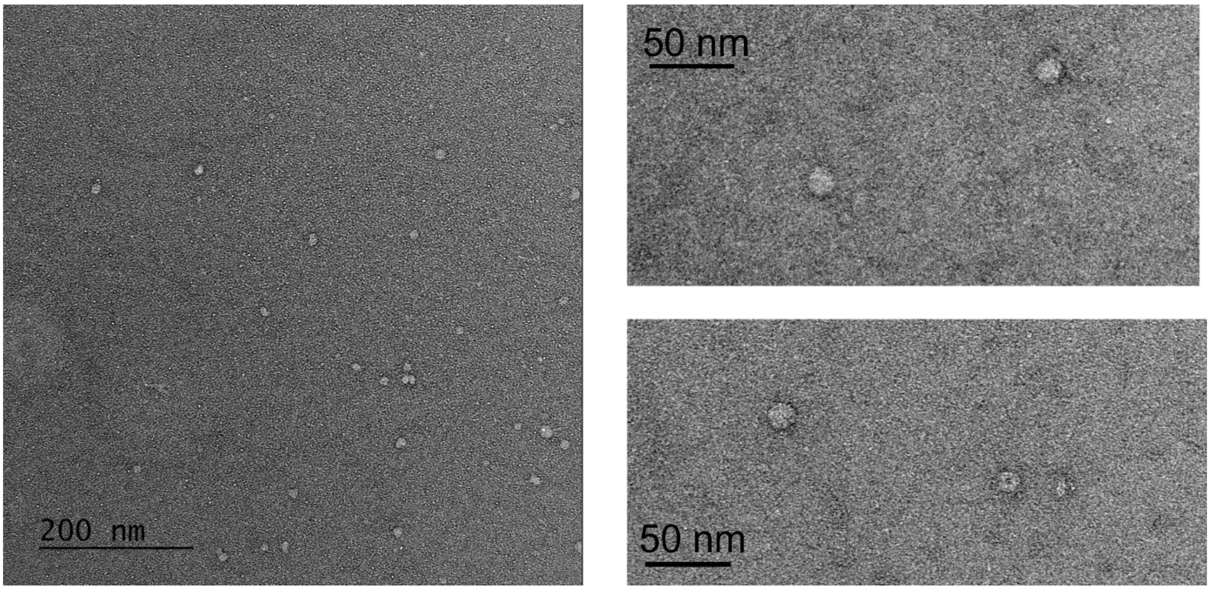

Supplementary Figure 1. BK-SMALPs can be visualised by negative stain electron microscopy. Representative images obtained when purified BKα+β1-SMALPs were immobilised on negatively charged copper grids and stained with 2% uranyl acetate. Grid was imaged on the JEOL2100+ TEM at 60,000 X magnification (at Warwick University Imaging Facility)

Supplementary Figure 2

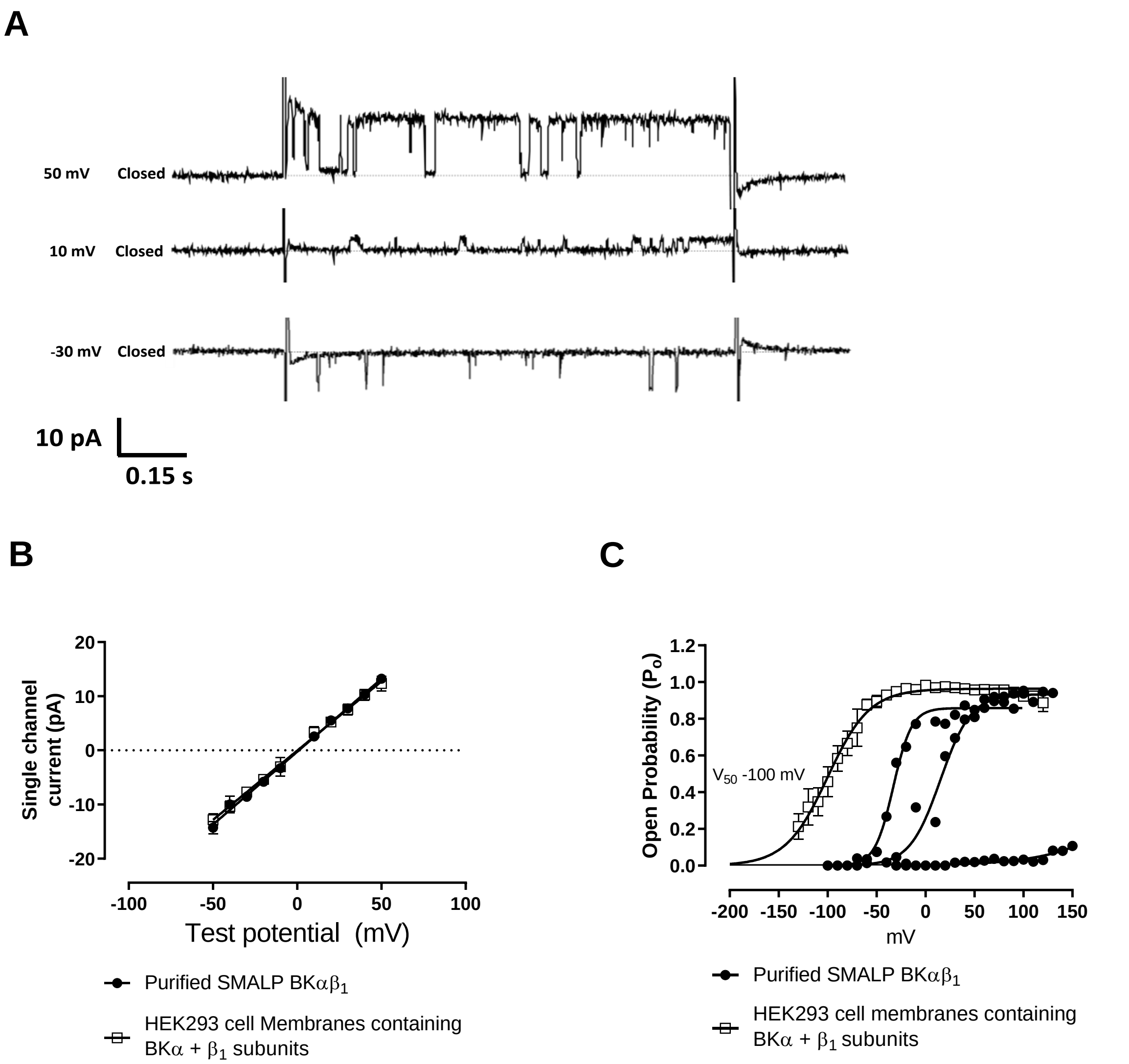

Supplementary Figure 2. BK channels (hslo α + β_1) purified using SMALPs can form functional BK channels in planar lipid bilayers, however voltage gating is modified. The recordings were made at a range of trans-bilayer potentials in the presence of 50 µM free Ca^{2+}. The planar lipid bilayer was formed from 50:50 POPS and POPE. (A) The BK α + β_1 channel/SMALP complex inserted forward into the bilayer because depolarisation increases the open probability and hyperpolarisation decreases the open probability. At -30 mV channel openings are brief whereas at + 50mV channel openings are long and channel closing is brief. (B) Single channel current amplitude for SMALP purified BKαβ (●) and those from membrane preparations containing the BKαβchannel (□). The single channel conductance is 269 ± 7 pS (n=3) for purified channels and 256 ± 4 pS (n=9) for channels from membrane. (C) Plots of open probability versus trans bilayer voltage for SMALP purified BKαβ (●) and those from membrane preparations containing the channel (□). SMALP purified channels show a reduced voltage dependent gating, compared to those from membrane preparations.

Supplementary Figure 3

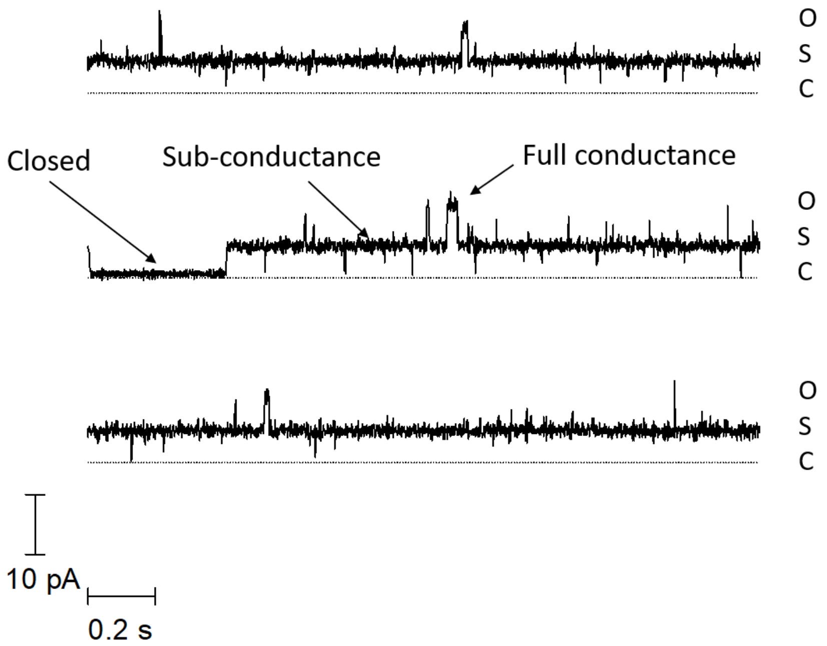

Supplementary Figure 3. BK channels (hslo α + β1) purified using SMALPs can form functional BK channels in planar lipid bilayers composed of positively charged lipids, however gating is modified. It was hoped that the inserted SMALP would disperse more readily, and the BK channel would gate more freely in a positively charged PLB. The recordings were made at + 50 mV in the presence of 50 µM free Ca^{2+}. The planar lipid bilayer was formed from 20:80 POPC and POPE. The channel inserted but has difficulty opening at + 50 mV. The channel spends a large part of its time in one of many sub-conductance states. C, S and O represent closed, sub- conductance and open states.
